# Tunable Microfluidic Devices for Hydrodynamic Fractionation of Cells and Beads: A Review

**DOI:** 10.3390/s151129685

**Published:** 2015-11-24

**Authors:** Jafar Alvankarian, Burhanuddin Yeop Majlis

**Affiliations:** Institute of Microengineering and Nanoelectronics, National University of Malaysia (UKM), 43600 Bangi, Selangor, Malaysia; E-Mail: burhan@ukm.edu.my

**Keywords:** tunable, microfluidic, mechanical, hydrodynamic, fractionation, microbeads, cells

## Abstract

The adjustable microfluidic devices that have been developed for hydrodynamic-based fractionation of beads and cells are important for fast performance tunability through interaction of mechanical properties of particles in fluid flow and mechanically flexible microstructures. In this review, the research works reported on fabrication and testing of the tunable elastomeric microfluidic devices for applications such as separation, filtration, isolation, and trapping of single or bulk of microbeads or cells are discussed. Such microfluidic systems for rapid performance alteration are classified in two groups of bulk deformation of microdevices using external mechanical forces, and local deformation of microstructures using flexible membrane by pneumatic pressure. The main advantage of membrane-based tunable systems has been addressed to be the high capability of integration with other microdevice components. The stretchable devices based on bulk deformation of microstructures have in common advantage of simplicity in design and fabrication process.

## 1. Introduction

Effective fractionation of beads and cells in microfluidic devices is essential for applications such as lab-on-chip for pharmaceutical and biological studies [1,2,3]. Functions such as separation [4], filtration [5], focusing [6], isolation [7], capturing or trap and release [8] have been implemented in various forms on bulk and single cell or bead using microfluidic platforms [9,10]. Different known properties and behaviors of the cells and beads in microfluidic systems are utilized in diverse sorting approaches [11,12,13]. The performance index of selectivity at different flow rates and concentrations has been defined and presented for characterization of microfluidic devices in fractionation of beads and cells [7,14,15,16].

The active techniques of fractionation that are reported extensively for microfluidic applications are normally based on discrimination between different parameters of cells or beads due to the physics of motion in fields of acoustic [17], optical [18,19], electrical [20], or magnetic [21,22] as external sources of forces. Some of these techniques need labeling of the cells and therefore they demand complicated, bulky, and costly systems for effective functioning [23]. The sorting performance of this class of microdevices is tunable for example by adjustment of parameters of the non-gravitational field of force [24].

The methods of hydrodynamic-based passively differentiate between the label-free microparticles only by using their intrinsic properties of size, form, deformability, stiffness, and viscoelastic behavior [13,25,26] in interaction with flow conditions and the internal structures of microdevices [27]. A variety of configurations of microchannels have been introduced for influencing the laminar flow and exploiting the inertial momentums of microbeads and cells at different throughput through the microfluidic devices for separation purposes [28,29,30]. A diverse class of microchannels and arrays of micropillars of various shapes have been designed for improvement of interaction of fluid steam with the microparticles [31]. In some cases, enhancement is achieved by activation of microstructure surface using chemical biomarkers [32]. The purely hydrodynamic-based platforms are less complex with simple mechanical design and low-cost development process compared to the active separation techniques [33].

The main feature of mechanically tunable separation systems is deformation of microstructures of a device under mechanical loads such as external forces of compress and stretch or internal pneumatic pressure for the purpose of influencing the interaction between microstructures and fluid flow of microbeads or cells. Until now, there are just a few microdevices that have been developed by implementing this concept for modification of selectivity of cells and beads. Meanwhile, the technique has been reported for similar microfluidic-based applications such as mechanically tunable optofluidic devices [34], mechanically tunable fluidic antenna [35], tunable microlens in microfluidic [36], tunable open-channel microfluidic [37], and mechanically switchable wetting on wrinkled elastomeric surface [38]. The centerpoint of all such systems has been set on using mechanical force for deformation of microstructures made partly or entirely from an elastomeric substrates such as polydimethylsiloxane (PDMS) [39].

In this review, the focus is on a group of microfluidic devices that effectively have adjustability performance by using mechanical actuators for hydrodynamic-based fractionation of cells and beads. The small modulus of elasticity of elastomeric materials in addition to appropriate design of microstructures in hydrodynamic-based separation of beads and cells allow such devices to have a tunable selectivity [40]. The motivation toward development of mechanically tunable microfluidic systems has been the adjustment of separation of microbeads and cells at different setting by simple, effective and low-cost methods [41]. The characteristic dimensions of beads and cell are considered as the main criteria in reports of size-based focusing, sorting, separation, isolation, trapping, immobilization, and handling of bead and cell in microfluidic systems [25]. Deformability of particles under investigation has substantial effects on the whole course of device design and manipulation performance [12].

The microfluidic devices in present review are classified according to flexibility in two groups of structural-based and membrane-based. The reviewed works, such as the tunable separation using pillar-based microfluidic [15,40,41], tunable cup-shaped structures for single/multiple cell/bead trapping [42], and tunable hydrophoretic separation [43], are set in the first group, in which the entire device is usually deformed for mechanical actuation. In the second group, the microfluidic device is deformed partially by applying external force [44] or internal pneumatic pressure [45,46] for deflecting a thin membrane-type microstructure for particle separation purpose.

The present paper is organized as follows. The effect of substrate type and loading techniques on deformation of microstructures in a microfluidic device is briefly reviewed in Section 2. The realized techniques of adjustable devices for fractionation of beads and cells are reviewed in Section 3 and Section 4. Two major design concepts are addressed here. In the first group, tuning has been achieved by bulk deformation of entire microfluidic device by applying an external force of stretch or compress. In the second type, membrane-based designs that use pneumatic pressure as the actuating force for tuning of fractionation of microbeads and cells are summarized. Finally, the concluding remarks for future research are summarized.

## 2. Mechanical Tunability

The forced deformation of microstructures in elastomeric microfluidic devices is an easy way of changing the width and height of microchannels in existing systems without repeating the costly fabrication process of a new design. Physically, when the microchannel dimensions are distorted under stress, the parameters of fluid flow such as pressure and velocity components that are important components of momentums of beads and cells are altered. The direction, frequency, and total amount of deformation of microstructures determine the amount of adaptation occurs to interaction between microbeads or cells with the fluid flow and the walls of modified microchannels [42]. The intensity of deformation and its precision that takes place in the microfluidic device depends on design of microstructures, mechanical property such as modulus of elasticity of substrate, and applied loads. The source of mechanical loads for actuating a microfluidic device can normally be a form of external force (tensile or compress) or internal pneumatic pressure.

### 2.1. Elastomeric Substrate

All materials utilized in fabrication of microfluidic devices exhibit a level of elasticity under different loading intensities. Those with large modulus of elasticity such as silicon (E = 169 GPa) [47,48,49] demonstrate very small strains even under very large stresses. The rubber-like polymers such as polydimethylsiloxane (PDMS) (E = 1.32−2.97 MPa) exhibit considerable elastomeric deformations even under small forces due to its low modulus of elasticity, a behavior that is highly favorable and critical for mechanically adjustable microdevices [50]. The stress–strain relationship is nonlinear in rubbers, especially at high strain conditions, but it has been demonstrated that at low stretch strains of less than 40%, a linear approximation well predicts the behavior and simplifies the analysis. The elastomeric microstructures are buckled only under compressing force if the stress increases from a critical value. The hard polymeric materials such as polymethylmethacrylate (PMMA) [51,52,53] (E = 2.38 GPa) or polyurethane methacrylate (hard-PUMA) [54] (E = 241 MPa) show a relatively high rigidity compared to elastomers to be used in mechanically adjustable devices. The soft-PUMA (E = 1.35 MPa) is another material with excellent elastomeric properties that has been introduced by the authors of present review to the research community for microfabrication and rapid prototyping of microfluidic devices [7,15,55,56,57].

### 2.2. Microstructure Design

**Figure 1:**
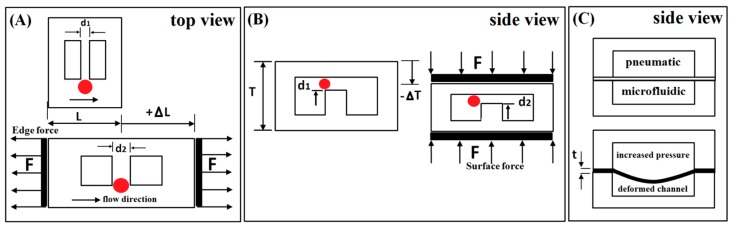
Schematic illustrations of the loading types for mechanical adjustment of microfluidic devices designed for size-based discrimination of microparticles. (**A**) The top view of microstructures of a device stretched by edge force for increasing the width of narrow gap; (**B**) Frontal view of a slit in a microchannel in a device compressed by surface distributed force for reducing the height of narrow gap; (**C**) Alteration of critical dimension of microchannel and cross-section area by elastic membrane deflected with pneumatic pressure.

The size-based hydrodynamic manipulation of beads and cells in microfluidics devices has been achieved by adjustment of a critical geometry such as the width of narrow gaps in microstructures or the cross-section profile of the microchannel that stream of micro particles has to pass through it. The mechanical tuning with high resolution of the manipulation in such systems has been conducted by elastic modification of the critical dimensions of microstructure defined based on characteristic size of the microparticles. As shown schematically in Figure 1, different techniques of loading such as compressing or stretching of the whole device has been used to effectively regulate the efficiency of a system via elastomeric deformation of microstructures. The required load and its direction are dependent on design of the microstructure, the modulus of elasticity of the substrate, and the target adjustments.

In Figure 1A, schematic of a channel is shown with critical dimension of d_1_ stretched to d_2_ by the force of F. The force is normal to the channel direction and uniformly distributed on the edges of device. This simple design has been implemented in tunable pillar-based system with uniform micron-scale spacing for size-based microfiltration on PUMA substrate [15] and tunable high-resolution deterministic lateral displacement (DLD) on PDMS [40]. The required force for bulk deformation of the microstructure which is defined by *F* is applied on the microchip by actuator clamped to the microdevice for stretching the length from *L* to *L +* Δ*L*. To have a micron precision tunability, actuating systems with high resolution have been used. The required external force in the device plane has been supplied by off-chip actuators that are either manually [40] or automatically [15] controlled.

In Figure 1B, the schematic drawing illustrates a microchannel cross-section under compressing force for changing the critical dimension of *d_1_* to *d_2_* while the overall thickness of the elastomeric device is reduced by Δ*T*. The concept of adjustable devices has been exploited for elastomeric tuning of hydrophoretic separation [43]. The force required for bulk deformation of microstructure is applied off-chip and distributed uniformly on top and bottom surfaces of the microchip. The nonlinearity in compressive deformation or buckling of PDMS microstructures has been reported in this type of device loading. The tuning techniques based on bulk deformation of the microdevice have the challenging point of integration with the other microfluidic components due to the bulky mechanical actuation systems.

Deflection of a thin membrane by pneumatic pressure is a well-known technique that has been implemented by several researchers for controlling the pneumatic and microfluidic zones in a microdevice for separation [46], isolation, or dynamic trap and release [8,58] of the beads and cells. The objective of membrane deflection is to reduce the effective height of the fluidic microchannel locally and therefore its hydraulic diameter or cross-section area for manipulation adjustment. Most of the reported works are based on using pulsing pneumatic or hydraulic pressure for actuating the elastomeric membrane in the device plane [45,47,58,59,60]. The PDMS has been the common substrate in fabrication of the thin flexible membrane or diaphragm with microscale thicknesses [8]. A schematic of such a microfluidic device is depicted in Figure 1C. The pressure difference between the zones of pneumatic and microfluidic flow in the microchannels, Δ*P*, determines the effective deflection of the membrane with the thickness of *t*. Design and sizing of the microfluidic device is normally performed such that any unwanted deformation in microstructures is at lowest while displacement of membrane is maximum [45,46].The pneumatic pressure or vacuum required for actuating the membrane at different frequencies is usually supplied by off-chip pumping systems [45]. The compatibility of this technique with other microfluidic components for on-chip integration has been demonstrated in various reported research works.

### 2.3. Device Tuning Methods

Several design models have been introduced based on manipulation of microbeads or cells in interaction with fluidic flow and device internal microstructures [27,61]. In hydrodynamic-based separations, the most important factors are contours of microstructures, flow regime, and mechanical characteristics of beads or cells [27]. The mechanical tuning of such devices by forced alteration of geometry of microstructure modifies the fluid flow and separation efficiency of microparticles [29]. The adjustments on microstructures can be static so that the force and strain are applied before starting of microfluidic testing [15,40]. In this technique, the actuation system is normally a separate mechanism from the microfluidic control while in dynamic tuning; the mechanical loading is simultaneous and integrated with the particles manipulation assembly [62,63]. Depending on design of the device and its structural elements for particles manipulation, the suitable and utilized techniques of adjustment of the microstructures are categorized in two types of bulk and local or membrane-based deformation.

## 3. Deformation of Microstructure Layer

**Figure 2:**
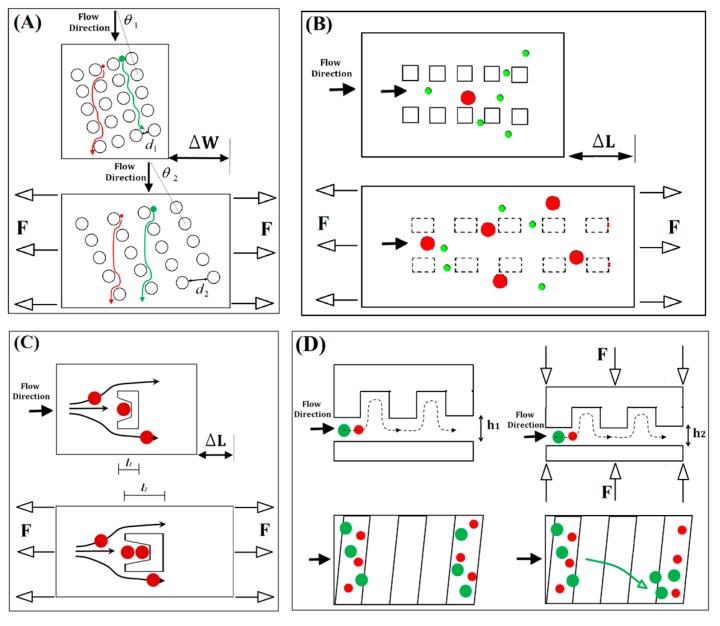
Schematic illustrations of reported tunable microfluidic concepts for separation of beads and cells by bulk deformation of microstructures under mechanical forces. (**A**) A design of microstructure for DLD-based separation; (**B**) A linear array of micropillars for size-based microfiltration; (**C**) Trapping of single or multiple cells using adjustable cup-shaped posts; (**D**) Hydrophoretic focusing by changing the microchannel height under compressive force.

**Figure 3:**
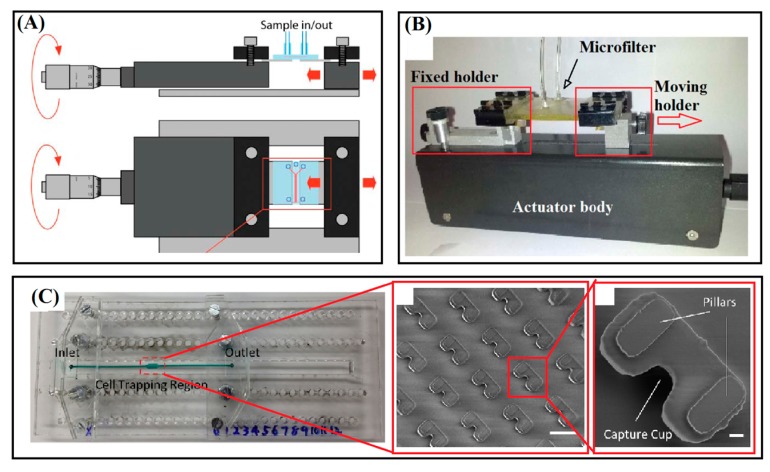
The mechanical actuation systems of tunable microfluidic devices for separation of microbeads and cells. (**A**) A modified micrometer stage for stretching a microstructure for DLD device. Image adapted from [40]; (**B**) A modified electric syringe pump for stretching linear arrays of micropillars in elastomeric PUMA device. Image adapted from [15]; (**C**) A setup fabricated for extension of array of cup-shaped microstructures in PDMS layer for trapping of controllable number of cells and beads. Images adapted from [42].

Physical manipulation of cells and beads by mechanical tuning of elastomeric microfluidic devices via bulk deformation of their microstructures has been implemented in different ways. As depicted schematically in Figure 2, by applying compressing or stretching forces and altering the overall size of width, length, or thickness of the microfluidic device in macroscale using mechanical systems as shown in Figure 3, a dimension of the structure in microscale which is critical to efficiency of the device has been adjusted at the target setting.

### 3.1. Separation by Arrays of Micropillars

The elastomeric microfluidic devices with pillar-based microstructures have been demonstrated as suitable candidates in tunability for separation of microbeads and cells. A microstructure layer consisted of an array of pillars is mechanically stretched to increase the inter-pillar spacings with micron or nano resolution. The limitation on tunability of the device is maximum force of actuator and stretchability of the substrate. The tuning is made before microfluidic injection and is reversible by the elastomeric material. The demonstrated advantage of such mechanisms has been the simplicity and low-cost fabrication process.

**Figure 4:**
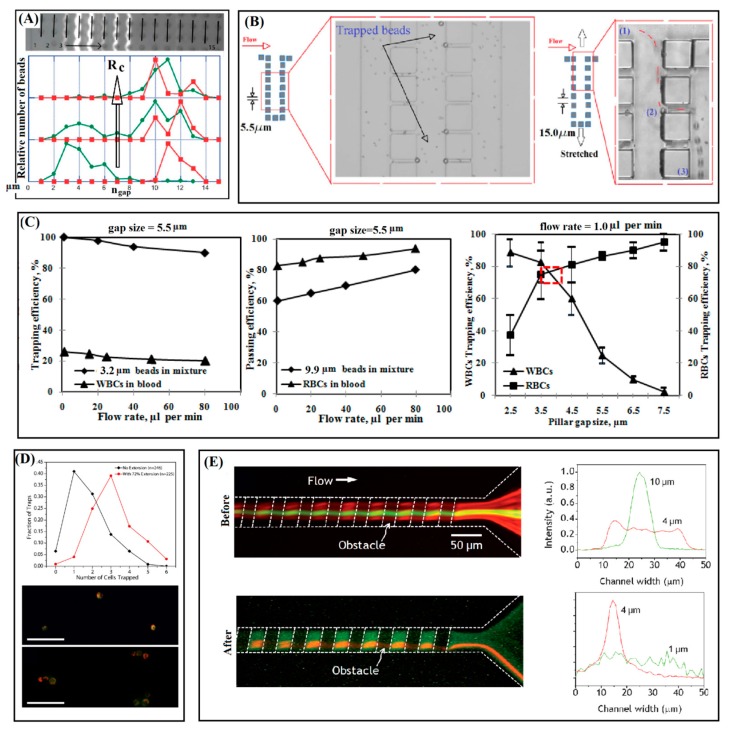
The reported results of device tuning for separation of cells and beads. (**A**) Separation of 5 µm from 8 µm beads using method of Deterministic Lateral Displacement tuned at different settings of critical radius. Image adapted from [40]; (**B**) Size-based filtration of microbeads of 3.2 and 9.9 µm using stretchable linear array of pillars at flow rate of 1.0 µL/min. Image adapted from [15]; (**C**) Effects of flow rate and gap size on separation of microbeads (3.2 and 9.9 µm) and blood cells (white and red) using stretchable linear arrays of pillars. Image adapted from [14]; (**D**) The number of trapped cells using tunable array of cup-shaped elastomeric pillars before and after stretch. Image adapted from [42]; (**E**) Top view of a microfluidic device for tunable hydrophoretic focusing of microbeads for before and after mechanical compression. Before deformation, a mixture of 10 µm and 4 µm beads at flow rate of 1.0 µL/min has been tested. After compression, a mixture of 4 µm and 1 µm beads at flow rate of 0.4 µL/min has been used. Image adapted from [43].

The mechanically tunable device based on DLD technique as shown schematically in Figure 2A has been reported by Tegenfeldt’s group for continuous flow separation [40]. This method works by discrimination between radius of the beads and a critical value as the tunable factor which is a function of parameters of *θ* and *d*, with resolution as low as 10 nm as depicted in Figure 2A [64]. Using the mechanical system with micrometer precision shown in Figure 3A, the PDMS microstructure has been stretched nonlinearly up to 150% of its original width. The critical radius has been adjusted from ~7.4 µm to ~9.0 µm for tuning of separation selectivity of 5 µm and 8 µm Polystyrene (PS) beads with flow velocity of ~500 µm/s. Effect of stretching the microstructure layer has been expressed by graphs of distribution number of the beads across the pillars array in Figure 4A. The beads with radius smaller than the critical value follow the main stream and the larger ones are guided to the direction of array of circular pillars. By increasing the critical radios, the distance between the peaks of distribution of beads is decreased as the tuning result for separation efficiency in the two-layer device.

Another tunable system is a work of the authors of the present review on using linear arrays of pillars with square cross-section for microfiltration of blood cells and beads [15]. The separation concept is according to size-based trapping of large beads or cells in arrays of micropillars with uniform and tunable spacing illustrated schematically in Figure 2B [7,14]. The tuning method is exploiting the nonlinearity behavior presents in the forced deformation of the arrays of pillars under stretch so that a small elongation of less than 20% on a monolithic two-layer device made entirely from PUMA is translated to three fold changing of the spacing of pillars as the critical dimension. The critical size with initially value of 5.5 µm has been tuned to 10.5 µm and 15 µm using a mechanical stretching assembly shown in Figure 3B for selective filtration of microbeads of 3.2 and 9.0 µm or whole blood cells, as shown in Figure 4B. A similar platform has been employed for optimization of efficiency and selectivity in microfiltration of blood cells and microbeads by the effects of flow rate and inter-pillar spacing (see Figure 4C) [14].

### 3.2. Cup-Shaped Elements for Trapping

The microfluidic devices consisted of array of cup-shaped elements as shown in Figure 2C has been reported by Lin’s group for trapping of variable number of cells using tunable depths [42] and for the depth-size effect on number of the trapped cells [65]. The PDMS layer has been casted using SU-8 mold and the device has been tested for trapping of MCF-7 cells. At unstretched condition, the cup-shaped pillars (width × depth, 29 µm × 25 µm) have trapped only one or two cells. After extension of the assembled device to a nonlinear level using the mechanical system shown in Figure 3C, the stretched cup-shaped elements (18 µm × 36 µm) were capable of trapping three cells while some percentage of them only trapped one or two cells as reported in Figure 4D.

### 3.3. Tuning of Hydrophoretic Effect

The hydrophoretic continuous separation of bulk of microparticles has been adapted tunable by Park’s group using elastic deformation of the microchannel cross-section [43]. The original technique that has been developed in the same group is based on the fact that the beads with diameters larger than one-half of the microchannel height follow a hydrophoretic separation path [66] while other beads follow a rotational flow. By mechanical compressing of the PDMS device under surface distributed force from top, the height of a microchannel with array of anisotropic obstacles is reduced so that the focusing criteria of hydrophoretic is changed and shifted to a smaller range of particle size as shown schematically in Figure 2D and the fluorescent images in Figure 4E.

## 4. Elastomeric Membrane Deformation

**Figure 5:**
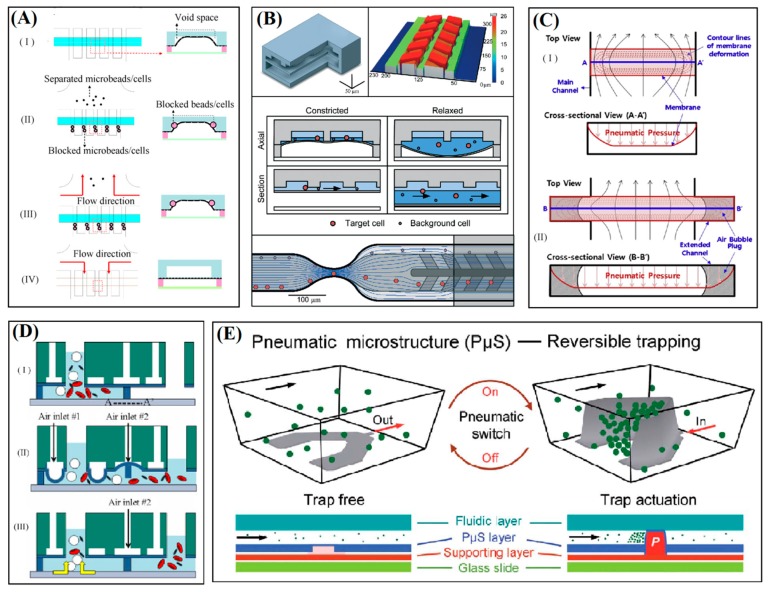
Schematic illustrations of concepts that have been used for membrane-based manipulation of cells and beads in microfluidic devices. (**A**) Blockage and release of microbeads at voids formed in the corners of microfluidic channel by pneumatically deflected membrane. Image adapted from [46]; (**B**) A resettable membrane-based trapping of cells and beads in microchannel with constrictions using a clog-free filtration technique. Image adapted from [16]; (**C**) A uniform slit is formed between deflected membrane and the microchannel by incorporating plugs of air bubble for enhancement of size-based filtration. Image adapted from [8]; (**D**) A pneumatically activated system for tuning of gap between a floating block and bottom of the microfluidic channel for size-based separation of blood cells. Image adapted from [45]; (**E**) A pneumatically activated microstructure for dynamic capturing and releasing of beads or cells in microfluidic elastomeric device. Image adapted from [58].

Mechanical tuning of microfluidic devices for the purpose of manipulation of beads and cells by technique of local deformation of thin membranes under pulsing pneumatic pressures or controlled by valve has been exploited by several researchers. A number of such novel devices have been introduced with PDMS membrane as the core element or for actuation of a microstructure for handling [67], trapping [62], trap and release [63] of single or bulk of cells or microbeads. In Figure 5, simple schematics illustrate different working concepts of such systems.

### 4.1. Blockage of Microchannel Cross-Section

A microdevice has been reported by Huang *et al.* for microfiltration using tunable blockage of a microchannel cross-section [46]. The device has four layers including a PDMS membrane with thickness of 40 µm, which is actuated by pneumatic pressures of 3–17 psi with pulsation of 1–16 Hz for separation of 5–20 µm beads with concentration of 2500 beads/µL. As Figure 5A shows, by different settings of the actuating pressure, the beads or cells larger than the critical size of the voids are trapped in two corners of the microfluidic channel while the smaller microbeads are passed through and collected in a filtrate reservoir. By releasing the pneumatic pressure, the membrane rest back to its initial position and the trapped beads are directed by a reversed flow to the collecting reservoir.

Another filtration mechanism consisted of resettable traps for cells and beads with selectivity based on size and deformability has been introduced recently by Ma’s group [16]. The initial concept of the device is about using oscillating membrane for chromatography of cells in a microchannel [68]. The flow and control layers of the device have been made by casting of PDMS of different ratios of base-to-crosslinker for different elasticity suitable for fluidic microstructures with low flexibility and pneumatically deflected diaphragm with high flexibility. The operation cycle of device is based on repeating steps of filtration, purging and collection. As Figure 5B shows, deflection of the diaphragm under positive pressure reduces the channel height so that only small beads or highly deformable oversize cells in the flow can pass through it. The percentage of tapped or passed particles of any size depends on the range of control pressure. The presented system has been analyzed for scalability, anti-clogging mechanism, as well as serial enrichment of rare cancer cells in leukocytes.

Additionally, a microfluidic device has been introduced for enhancement of separation efficiency in a straight microchannel integrated with membrane. Air bubble plugs are incorporated for formation of a wide and uniform slit. The membrane with thickness of 50 µm is actuated by pneumatic pressures of 50–80 kPa in a three-layer device [8]. As schematic of the concept in Figure 5C shows, the key point of the technique is exploiting the air bubble plugs for increasing the blockage of the beads with flow rate of 10–20 µL/min and concentration of 10 beads/µL in the microchannel. The increment of trapping efficiencies for the cases of with an air plug compared to without it is reported as substantial for tested flow rates and bead size.

### 4.2. Actuation of Floating Microstructure

A microfluidic system has been introduced for size-based separation of microbeads and blood cells by Lee’s group that works using membrane deflection as the actuating element for a floating block structure [45]. The critical parameter for size-based discrimination of cells or beads is the gap between microchannel floor and a movable block as shown in Figure 5D. The gap size is set in the range of 1–13 µm by pulsing pneumatic pressures of 0–7.2 psi at optimum flow rates of 21.4 and 3.0 µL/min for separation of blood plasma and red blood cells. The trapped beads or cells are removed from the filtration zone by a back-flush after the membrane is released to its initial form. The microfilter, required microfluidic micropumps along with the reservoirs are all integrated in one chip. Similarly, a system of multiple barriers integrated on membrane has been used for tunable cell handling of nanoliter volumes of whole blood samples [67].

### 4.3. Dynamic Cup-Shaped Elements for Trapping

Recently, a microfluidic device has been developed for trapping of controllable number of cells by Liu *et al.* using array of dynamic U-shaped microstructures [58]. One of the main advantages of this new idea over the similar device with the array of static cup-shapes is actuation by membrane instead of bulk deformation of microstructures [42]. As shown in Figure 5E, physical form of the microstructures and the three modes of non-touch, local touch, and total touch have been interestingly realized by changing the shape of 18 µm thick PDMS membrane in the four-layer device that is mechanically operated by air pressures of 0–20 psi. It has been demonstrated that performance of the mechanism for reversible and controllable trapping of living mammalian cells are highly depends on the microfluidic flow rate (0–200 µL/min), actuating pressure and seeding time (0–15 min).

## 5. Conclusions

So far, several elastomeric microfluidic devices have been designed and fabricated with mechanical tunability for size-based fractionation of cells and beads with applications defined in biological studies and other research fields. Tunability of the described systems enables us to adjust and to optimize the performance and efficiency of operation in microscale for the working conditions suitable for each specific application. In Table 1, the reported tunable microfluidic systems are summarized for comparison based on their adjustable geometry, application, and development challenges.

**Table 1:** Summary of demonstrated performance of tunable elastomeric microfluidic devices for size-based selectivity in separation of cells and Polystyrene beads.

Method	Tunable Geometry and Actuation	Application and Flow Conditions	Development Challenges
Stretchable DLD [40]	variable inter-pillar spacing and discrimination resolution of 10 nm	continuous fractionation, beads (5 and 8 µm), flow rate of 500 µm/s	stretcher integration to complex systems, non-uniformity of strains, stick-slip behavior
Stretchable pillar-based [15]	inter-pillar spacing of 5.5–15 µm and actuator resolution of 0.165 µm	Microfiltration beads (9.9 and 3.2 µm, 50/µL and 50,000/µL) and blood cells, flow rate of 1.0 µL/min	stretcher integration to complex systems
Stretchable pillar-based [14]	inter-pillar spacing of 2.5–7.5 µm and actuator resolution of 0.165 µm	microfiltration optimization, beads (9.9 and 3.2 µm, 50/µL and 50,000/µL) and blood cells, flow rates of 1.0–80 µL/min	stretcher integration to complex systems
Tunable hydrophoretic [43,66]	obstacle gap for hydrophoretic criterion adjusted on 7.0–2.5 µm	continuous focusing, beads (mixtures of 10,4, and 1 µm, 20, 7.3, and 1.8 × 10^2^/µL), flow rates of 0.4 and 1.0 µL/min	nonuniform deformation of microchannel cross-section under mechanical press
Stretchable cup-shaped structures [42]	depth of elastomeric structures stretched to 79% of initial value	device modulation for number of trapped cells cancer cells (MCF-7, 1000/µL), flow rate of 10 µL/min	nonlinear deformation, stretcher integration to complex systems
Dynamic cup-shape structures [58]	controllable dynamic array of U-shape structures, pneumatic pressures of 0–20 psi	trap and release for patterning and manipulation of human cells, (A549, HepG2, MCF-7, 5000/µL), flow rates of 0–200 µL/min	typical in microfluidics
Channel cross-section corners [46]	corner voids of channel blocked by membrane to 5 µm, pulsing pneumatic pressures of 3–17 psi at 1–16 Hz	filtration and recovery, beads (5–20 µm, 2500/µL) and cells (chondrocytes), flow rates of 3.3–14.9 µL/min	typical in microfluidics
Floating block [45]	floating block forms narrow gap size of 1–13 µm, pneumatic pressures of 0–7.2 psi and pulsation of 1–11 Hz	separation, beads (1.0, 4.8, 10 µm and concentration of 16.63, 4.25, and 0.26 × 10^3^/µL) and blood cells	typical in microfluidics
Resettable trap [16]	diaphragm deflection for size discrimination of <1 µm, pneumatic pressures of 0–5.8 psi	filtration and recovery by size and deformability, beads (6.4, 7.3, 9.5, 10.1 µm) and rare cancer cells from blood (UM-UC13, 1/1000 leukocytes), flow rate of 4–6 mm/s and 15,000 cells/min	typical in microfluidics

The microfluidic systems that are adapted tunable using existing methods such as DLD [64], pillar-based [7], hydrophoretic [66] and cup-shaped structures [65] have clear similarity in microfluidic characteristics with the original platforms in quantitative terms of effective flow rate, cell/bead throughput, cell/bead concentration and separation efficiency. By considering that the architecture of microstructures in the adapted and the original device is equal; both groups have the same basic advantages and weaknesses regarding separation of beads or cells. The main difference between performances of the original device and its tunable modified version is the range of selectivity of cells or beads following adjustment of critical geometries. These microfluidic devices are adjustable by bulk deformation of the device layers and offer advantage of less fabrication steps compared to other tunable systems. Several challenges have been addressed mostly based on the reported fabrication processes and experimental works; however, the most significant can be the integration of microfluidic device and the mechanical actuator to more complex microsystems. Among the methods of tunable DLD, pillar-base, and hydrophoretic, it has been reported that the deterministic lateral displacement has the highest demonstrated tunability resolution in size-based fractionation of cocktails of cells or beads.

The two methods of stretchable and dynamic cup-shape structures that work base on modified forms of fixed U-shape posts for cell trapping, demonstrate the importance of novel ideas in development of alternative devices with similar applications and general performances but with more flexibility and compatibility with other microfluidic components.

The membrane-based tunable devices are designed with clog-free mechanism, which is very important for continuous working and integration with other microfluidic components such as micropumps and valves for applications in more complex systems and lab-chip devices. These types of microfluidic systems have more creative designs for tunability compared to other devices. The materials and processes necessary for development of these devices are typical in microfluidic technology.

All reviewed tunable hydrodynamic-based microfluidic devices are potentially applicable for separation of solid microbeads and deformable biological cells. This review article provides a good reference for investigation of the microfluidic platforms with purely mechanical tunability and the corresponding limitations and possibility of further development and enhancement.
